# Efficacy and Safety of a Natural Remedy for the Treatment of Gastroesophageal Reflux: A Double-Blinded Randomized-Controlled Study

**DOI:** 10.1155/2016/2581461

**Published:** 2016-10-12

**Authors:** Umberto Alecci, Francesco Bonina, Andrea Bonina, Luisa Rizza, Santi Inferrera, Carmen Mannucci, Gioacchino Calapai

**Affiliations:** ^1^Italian College of General Practitioners and Primary Care, Florence, Italy; ^2^Department of Drug Sciences, University of Catania, Catania, Italy; ^3^Bionap srl, R&D Contrada Fureria, Piano Tavola, 95032 Belpasso, Italy; ^4^Department of Biomedical, Dental Sciences and Morphofunctional Imaging, University of Messina, Messina, Italy

## Abstract

Gastroesophageal reflux (GER) is a common, chronic, relapsing symptom. Often people self-diagnose and self-treat it even though health-related quality of life is significantly impaired. In the lack of a valid alternative approach, current treatments focus on suppression of gastric acid secretion by the use of proton pump inhibitors (PPIs), but people with GER have a significantly lower response rate to therapy. We designed a randomized double-blinded controlled clinical study to evaluate the efficacy and the safety of a formulation based on sodium alginate/bicarbonate in combination with extracts obtained from* Opuntia ficus-indica* and* Olea europaea* associated with polyphenols (Mucosave®;* verum*), on GER-related symptoms. Male/female 118 (intention to treat) subjects with moderate GER and having at least 2 to 6 days of GER episodes/week were treated with* verum* (6 g/day) or placebo for two months. The questionnaires Gastroesophageal Reflux Disease-Health-Related Quality of Life (GERD-HRQoL) and Gastroesophageal Reflux Disease Symptom Assessment Scale (GSAS) were self-administered by participants before the treatment and at the end of the treatment.* Verum* produced statistically significant reduction of GERD-HRQoL and GSAS scores, −56.5% and −59.1%, respectively, in comparison to placebo. Heartburn and acid regurgitation episodes for week were significantly reduced by* verum* (*p* < 0.01). Results indicate that Mucosave formulation provides an effective and well-tolerated treatment for reducing the frequency and intensity of symptoms associated with gastroesophageal reflux.

## 1. Introduction 

Gastroesophageal reflux (GER) is a chronic, relapsing symptom that carries a risk of significant morbidity from resultant complications. However, many persons self-diagnose it and self-treat it not seeking medical attention for it and related symptoms. GER is due to the passage of gastric content into the esophagus [1]. It is a common condition and its prevalence in the general population has been evaluated to be about 5% in Asian countries and from 10 to 20% in Western countries [2]. Most of people with GER fall into 1 of 2 categories: those with nonerosive reflux or those with erosive esophagitis [3]. Nonerosive reflux has been commonly defined as the presence of classic gastroesophageal reflux in the absence of esophageal mucosal injury [4].

A large body of evidence has shown that health-related quality of life in people with GER is significantly impaired. A decrease in productivity and overall well-being is generally reported. Impairment caused by GER is comparable to, and sometimes is greater than, that observed in other chronic conditions, such as diabetes, arthritis, or congestive heart failure [5]. Impaired aspects of quality of life are disturbed sleep, reduced vitality, generalized body pain, unsatisfactory sex life, and anxiety. Nocturnal symptoms caused by reflux appear to have a particularly marked influence on quality of life and the burden of illness imposed by GER also has an impact on work productivity [6]. GER not only produces gastrointestinal symptoms, but it can be responsible for respiratory symptoms, because also respiratory problems, such as recurrent respiratory infections, persistent cough, life-threatening apneic episodes, and respiratory failure during fairly minor respiratory infections, can occur [7]. The typical symptoms, including heartburn and regurgitation, occur both during the day after meals and the night, when they frequently wake people up from sleep. In some cases, due to the worsening of symptoms, strictures can form leading to dysphagia [8, 9].

Current pharmacological treatments for GER focus on the suppression of gastric acid secretion by the use of proton pump inhibitors (PPIs) [10]. While effective for many patients, 30–40% of patients receiving medical therapy with PPIs experience troublesome breakthrough symptoms, and recent evidence suggests that this therapy is related to increased risk of complications [11]. People with GER have a significantly lower response rate to proton pump inhibitor (PPI) therapy, and consequently they constitute the majority of the refractory heartburn group [3]. However, the majority of people with symptomatic reflux requiring medical intervention are managed by their family physicians with PPIs in the lack of a valid alternative approach [6]. In the light of the above description of the state of art of GER management, we designed a randomized double-blinded controlled clinical study to evaluate the efficacy and the safety on persons affected by GER of a formulation based on sodium alginate/bicarbonate in combination with extracts obtained from* Opuntia ficus-indica* and* Olea europaea* associated with polyphenols, on GER-related symptoms. This formulation is based on information obtained from scientific literature describing that* Opuntia* species are having gastroprotective activities as demonstrated in various experimental models [12] and that* Olea europaea* leaves extract administration is able to prevent experimental formation of gastric lesions induced by stress [13]. Other studies demonstrated that sodium alginate [14] was effective in the treatment of GER and that an alginate-antacid formulation containing sodium alginate and sodium bicarbonate was safe and noninferior to omeprazole in achieving a 24-hour heartburn-free period in patients with moderate GER disease (GERD) [15].

As the medical device contains sodium alginate/bicarbonate and natural compounds found in foods, this formula presents no or fewer adverse effects than medications currently used in clinical medicine. Anyway, during the study we did not detect any potential adverse effects.

## 2. Methods

Study population included male or female subjects with at least 2 to 6 days of GER episodes per week, with heartburn, and with or without regurgitation, not taking alginate/antacid or PPIs treatment for at least the preceding 2 weeks, and able to understand the study and to complete the self-administered questionnaires. Prevalence estimates show considerable geographic variation, but median prevalence of subjects suffering from moderate gastroesophageal reflux in the general population is about 20% [5]. The sample size was calculated in order to observe a difference of 30% in the proportion of patients diagnosed with moderate gastroesophageal reflux treated for such symptom and the group nontreated (placebo), considering *α* = 0, 05, and *β* = 0.80 (the minimum number of patients required is *N* = 50 for each group). Apparently healthy male and female 118 subjects aged between 36 and 64 (median  age = 50) years affected by symptoms of GER but scoring not more than 3 points after administration of the GERD-HRQoL (Health-Related Quality of Life) questionnaire were recruited by 10 different general practitioners coordinated by a principal investigator according to the inclusion and exclusion criteria. Subjects were excluded if affected by diagnosed erosive esophagitis, Barrett's esophagus, and severe diseases such as asthma, cardiac, renal, metabolic, and tumoral pathologies. Exclusion criteria were also atypical digestive or extra-digestive symptoms without heartburn; gastric or duodenal ulcer; and history of upper digestive tract surgery or of upper digestive tract.

People selected by inclusion and exclusion criteria were enrolled after reading the Declaration of Helsinki. All subjects got oral and written information about the trial and gave individual written informed consent to participate before inclusion in the trial.

The trial ran from the beginning of March 2016 to the end of June 2016 and respected the ethical principles of the Seoul revision (2008) of the Helsinki Declaration and Good Clinical Practice and according to EN ISO 14155-1:2009 for medical devices. The study protocol received approval by the Ethical Committee of the Azienda Policlinico Universitario “G. Martino” of Messina on November 23, 2015, protocol number 79/15.

### 2.1. Screening and Randomisation

At screening (visit 1, day 60) after giving informed consent, subjects underwent medical history, physical examination, and vital signs. All subjects got oral and written information about the trial and gave written informed consent to participate before inclusion in the trial. Included subjects were randomly allocated to one of two groups treated as follows: Group 1, *N* = 59 to be treated with Mucosave sachet (*verum* 6 g/day); Group 2, *N* = 59 to be treated with placebo sachet (6 g/day). Randomisation by blocks of 3  (2 + 1) was double-blind. Successive blocks were balanced by 2 s. Neither subjects recruited for the study nor investigators were able to differentiate the two different treatments. Each single Mucosave sachet contained 4350 mg of maltodextrin, 500 mg of sodium alginate, 400 mg of Mucosave, and 300 mg of sodium bicarbonate; placebo was composed only of maltodextrin. Mucosave (Bionap srl, Italy) is a solid blend of extracts from* Opuntia ficus-indica *cladodes (32–35% w/w) and* Olea europaea* (olive) leaf extract (23–25% w/w); maltodextrin 40–45% w/w was used as technical support. As reported in the technical product information, the ingredient contained 3.7–4.3% w/w of total polyphenols (as luteolin 7-O-glucoside) verified by high pressure liquid chromatography (HPLC) method.

At screening (visit 1, day 60) after giving informed consent, subjects underwent medical history, physical examination, and vital signs. One hundred and twenty-six (*N* = 126) subjects were screened but 8 subjects failed to be included. Subjects included were treated with* verum *or placebo for two months. After 60 days' treatment, at visit 2, the questionnaires were self-administered again. Symptoms of GER were assessed using the GERD-HRQL, a validated questionnaire, at the screening visit. The questionnaire provides a composite score as well as an assessment of individual symptoms. The GERD-HRQL questionnaire assesses heartburn severity in nine questions using a scale of 0 (no symptoms) to 5 (incapacitating). This validated instrument includes six heartburn-related items and questions relating to other GERD symptoms, medication use, and satisfaction with present condition. The total GERD-HRQL score ranges from 0 to 50, with a higher score indicating more severe symptoms. The GERD-HRQL was developed to survey symptomatic outcomes and therapeutic effects in patients with GERD [9, 16]. The scale has 11 items, which focus on heartburn symptoms, dysphagia, medication effects, and the patient's present health condition. The GERD-HRQL takes approximately one minute to complete. Each item is scored from 0 to 5. We recruited only subjects obtaining a score between 2 and 3 corresponding to persons with symptoms noticeable and bothersome but not every day (score = 2) and those subjects suffering of bothersome symptoms every day (score = 3). In this way, subjects suffering from symptoms affecting daily activity and/or symptoms that are incapacitating to do daily activities, corresponding to subjects affected by overt GER disease, were excluded [9].

To assess the effectiveness of the product object of the study the Gastroesophageal Reflux Disease Symptom Assessment Scale (GSAS) was also self-administered. The GSAS is a self-administered questionnaire that asks patient to report the frequency, severity, and degree of bother during the previous week for 15 specific symptoms: heartburn or a burning pain inside chest or breast bone, a feeling of pressure or discomfort inside chest, food coming back into mouth, an acid or sour taste in the mouth, frequent gurgling in stomach or belly, feeling of pressure or lump in throat, nausea, burning pain in throat, bloating, belching, flatulence, feeling full after eating little, bad breath, coughing, and hoarseness. Scoring of the GSAS distress subscale is based on the presence of the symptoms and their bother ratings. Specifically, participants first indicate whether they had the symptom in the past week. If they did not have the symptom, then their score for the symptom is 0. If they did have the symptom, they then report how bothered they were by it on a 4-point scale (0 not at all, 1 somewhat, 2 quite a bit, and 3 very much) [17]. GERD-HRQL and GSAS were self-administered before the treatment (people enrollment) and at end of two months' period treatment.

### 2.2. Adverse Events

Adverse events (AEs) were collected at the two study visits (day 0 and day 60). An AE was defined as an untoward medical event that occurred during the study period, whether or not related to the study procedure or study products. Severe AE (SAE) was defined as an untoward medical event that resulted in death, was life-threatening, required inpatient admission or prolongation of hospitalization, or resulted in severe or persistent disability or incapacity.

### 2.3. Statistical Analysis

All results are expressed as means ± standard deviation (SD). Statistical significance was tested by unpaired Student's *t*-test, or Mann-Whitney *U* test, as appropriate. *p* < 0.05 was regarded as significant.

## 3. Results

**Figure 1:**
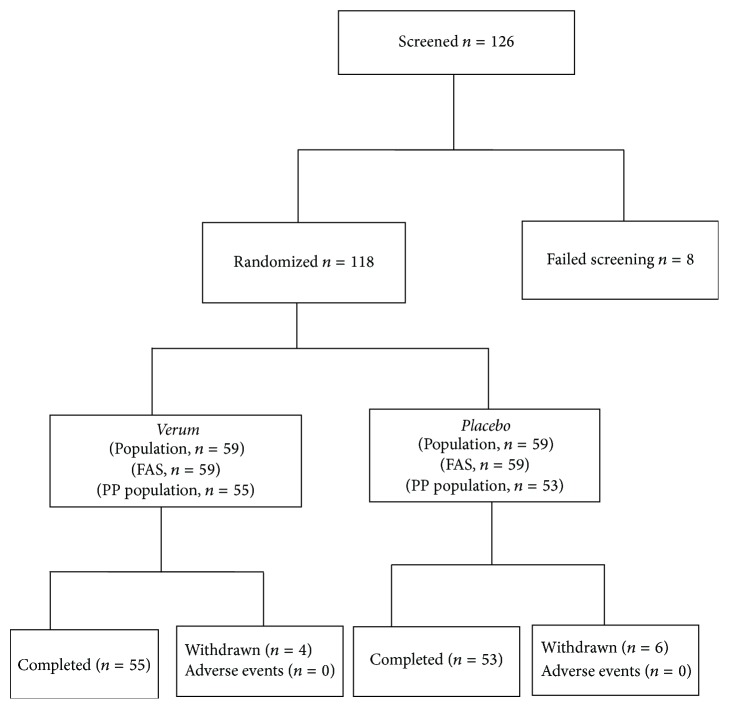
Flow diagram for the study (FAS: full analysis set; PP: per protocol).

Figure 1 shows the flow diagram of the study: 126 subjects were screened, 118 of which were recruited and randomized and divided into two groups, 1 and 2. The demographic characteristics of the two groups, 1 and 2, of the study including age, sex, body mass index, and smoking habit are shown in Table 1. Fifty-five and fifty-three subjects, respectively, of Groups 1 and 2, completed the study (Figure 1). There were no significant differences in any of these parameters between Groups 1 (treated) and 2 (placebo). Severity of symptoms was assessed immediately before treatment (baseline) and after 8 weeks of treatment.

**Figure 2:**
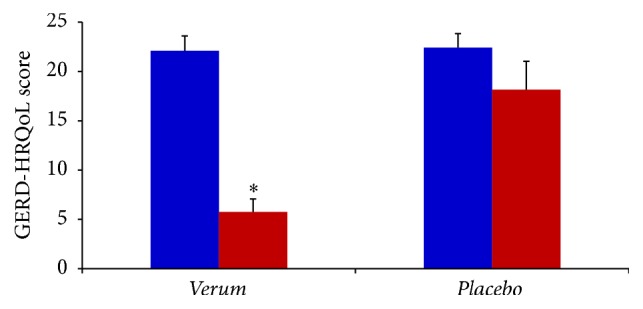
GERD-HRQoL score in subjects with GER before (blue) and after (red) 60 days' treatment with* verum* (*N* = 55) or* placebo* (*N* = 53). ^*∗*^ *p* < 0.05 versus red* placebo*.

**Figure 3:**
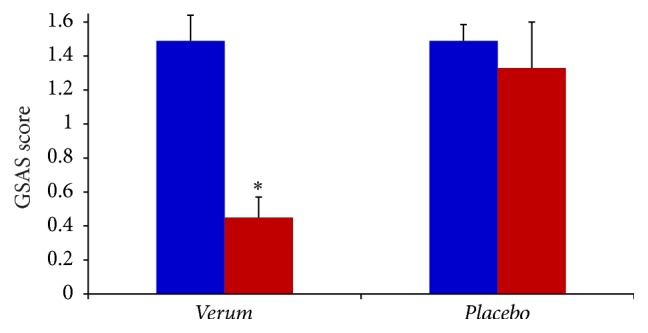
GSAS score in subjects with GER before (blue) and after (red) 60 days' treatment with* verum* (*N* = 55) or* placebo* (*N* = 53). ^*∗*^ *p* < 0.05 versus red* placebo*.

The baseline characteristics of all the evaluated GER-associated symptoms were similar in the two groups. Answers purchased by recruited subjects to the GERD-HRQoL questionnaire at the first visit (baseline before treatment) obtained the following basic score: Group  1 = 22.09 ± 1.5 and Group  2 = 22.41 ± 1.4. After two months' treatment Group 1 and Group 2 subjects answered the GERD-HRQoL questionnaire producing the score of 5.76 ± 1.3 (*p* < 0.01) and 18.15 ± 2.9 (*p* < 0.05), respectively (Figure 2), thus, showing that treatment with* verum* significantly produced in Group 1 reduction of symptoms associated with GER in the percentage of 74.3%. In the placebo group (Group 2) significant reduction of GERD-HRQoL was only 17.8%. Comparing the two scores obtained after two months' treatment and subtracting the percentage of reduction of placebo group (Group 2), it can be observed that treatment with* verum* produced a percentage of reduction of GERD-HRQoL score of 56.5% in subjects with GER. With analysis of data collected with GSAS questionnaire, a scale GSAS is sensitive to changes in severity of GER symptoms, showing similar results. Evaluation with GSAS indicates that treatment with* verum* significantly reduced the score obtained by Group 1 at the first visit in the percentage of 69.8% (*p* < 0.01). Placebo treatment reduced the GSAS score in the percentage of 10.7%, thus subtracting the placebo effect where it may be observed that* verum* treatment reduced GSAS score in the percentage 59.1% (Figure 3).

**Figure 4:**
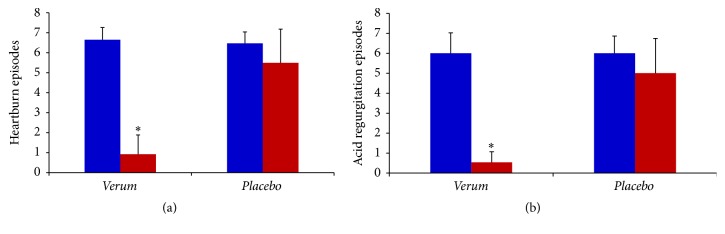
Heartburn and acid regurgitation episodes per week in subjects with GER before (blue) and after (red) 60 days' treatment with* verum* (*N* = 55) or* placebo* (*N* = 53). ^*∗*^ *p* < 0.05 versus red placebo.

Episodes for week of heartburn and acid regurgitation, the two main symptoms of GER, were both significantly reduced by treatment with* verum* (Figures 4(a) and 4(b)). No subject of Group 2 showed total absence of these two symptoms. During the treatment period no adverse event has been reported. Results of intention to treat analysis performed after preprotocol analysis confirmed the effectiveness of treatment with Mucosave in reducing symptoms associated with GER in this study.

## 4. Discussion

Epidemiological studies show that, even though gastroesophageal reflux adversely affects health-related quality of life, the majority of people with typical reflux symptoms have no evidence of erosive esophagitis at endoscopy [18, 19]. Current pharmacological treatments for GER are based on the suppression of gastric acid secretion by the use of PPIs. However, these drugs are effective in most persons with GER, where approximately 20–30% continue to experience reflux symptoms despite PPI treatment [10]. PPIs are the first-line choice in both reflux esophagitis and nonerosive GER. These drugs effectively inhibit the duration and extent of gastric acid secretion and provide more complete remission of the symptoms of heartburn than other forms of acid-suppressant therapy [20, 21]. However, the response to PPIs in people with nonerosive GER is less efficacious when compared with patients with erosive GER [22]. To reduce the intensity of the pivotal symptoms of GER, a formulation with alginate/bicarbonate,* Opuntia ficus-indica* extract, and* Olea europaea* (olive) leaf extract was developed and its effects were investigated in the present study.

The GSAS and GERD-HRQoL scales are reliable indicators of symptom distress and general QOL in people with GER. Symptoms associated with GER vary over time making it difficult to assess them and also remedies' effects in a constant and reliable way. This problem has been faced with short questionnaires that are administered in short time intervals. GSAS is considered the most comprehensive evaluative symptom scale so far. Besides GER-specific symptoms, it also focuses on associated symptoms (i.e., frequency of episodes, intensity of symptoms, and level of distress) [17, 23]. However, it is not fully believed that GSAS is a useful instrument for evaluation of multidimensionality of health-related quality of life in people with GER [24]. For this reason, we used also the more specific GERD-HRQoL scale. Furthermore, the strength of the GERD-HRQL scale is its sensitivity to responsiveness to the effect of treatments. In other words, the sensitivity is considered an ability of the scale to detect changes over time, important in assessing the efficacy of treatments [25].

The typical symptoms of GER include heartburn and acid regurgitation, occurring both during the night, frequently waking up from sleep, and also during the day, associated with meals. Since these symptoms have a great impact on quality of life, we extrapolated from GSAS questionnaire the answers of subjects corresponding to these items.

The results of the present randomized-controlled trial study show the efficacy of a medical device composed of alginate/bicarbonate,* Opuntia ficus-indica*, and* Olea europaea* extracts in reducing the common symptoms associated with GER. The study also shows that the product object of the study was safe and well tolerated for all the period treatment.

Alginate/antacid is a combination used in several self-medication products aimed for the management of GER symptoms, acting by a mechanical way at the gastric level. When it encounters low pH gastric content, it is able to create a physical raft on top of the gastric juice, counteracting its reflux. In several* in vitro* and clinical trials, alginate/antacid effect has been already evaluated in GERD management alone or in coadministration with active compounds [26–28]. Moreover, floating alginate/antacid system has been used as carrier for probiotic, drug, or plant extracts [29–31].

In this study, a combination of alginate/bicarbonate and two herbal gastroprotective extracts has been evaluated in human subjects in the management of GER discomfort. Beneficial effects in upper-gastrointestinal discomforts of the* Opuntia ficus-indica* cladodes and* Olea europaea* leaf extracts have been already documented in scientific literature [32–34].

*Opuntia ficus-indica *is a plant belonging to the Cactaceae family growing in semiarid areas of countries around the world and is mainly cultivated in the Mediterranean region and in Central America. Both fruits and cladodes (branches transformed taking the shape of leaves) have been used in traditional medicine in many countries [35]. In addition, in-depth studies on the biological activity of this plant have shown the antiulcerogenic activities. An involvement of* Opuntia ficus-indica* mucilages has been hypothesized, mainly formed by arabinogalactan and galacturonic acid, forming a defense layer in these gastroprotective effects [32, 33].* Opuntia ficus-indica* mucilage is strongly viscous which because of the negative charges causes strong intermolecular repulsion, resulting in expansion of the molecules. It is believed that this changing in molecular shape could be responsible for the protection of the gastric mucosa [34].

The leaves of* Olea europaea* plant have been widely used as traditional remedies in European and Mediterranean countries such as Greece, Spain, Italy, France, Turkey, Israel, Morocco, and Tunisia. They have been used as part of the diet in the form of extract, herbal tea, and powder containing potentially bioactive compounds with antioxidant and anti-inflammatory activities [36]. The extract of olive leaves has antioxidant and anti-inflammatory activities that have been shown in the laboratory animal. Studies demonstrated that water extracts of* Olea europaea* are capable of reducing levels of tumor necrosis factor-alpha in an experimental model wherein inflammation is induced by the treatment with lipopolysaccharide [37]. Moreover, it was also observed how the administration of extract of olive leaves is able to prevent the formation of gastric lesions induced by stress in an experimental model in the rat [13].

Olive leaf extract possesses antioxidant properties, which can positively influence gastroprotection. The main iridoide monoterpene oleuropein contained in olive leaf was usually thought to be responsible for pharmacological effects but it was recently observed that olive leaf is as a stable source of bioactive flavonoids. In fact, the contribution of flavonoids to the overall radical scavenging activity of olive leaf extracts has been investigated and luteolin 7-O-glucoside was found to be one of the dominant scavengers (8–25%) [38]. Finally, from leaves of* Olea europaea* the antioxidant hydroxy-phenyl-ethyl alcohols (hydroxyl-tyrosol and tyrosol), main components of olive, and the anti-inflammatory agent nocellaralactone and the flavonoidic aromadendrine have been isolated [39, 40]. These compounds probably contribute to the beneficial effects of* Olea europaea* observed in gastroesophageal reflux. Preclinical studies have been shown as olive leaf extract produces protective effects against stress-induced gastric mucosal damage by cold restraint stress in rats and these effects are associated with decrease in malondialdehyde (index of lipid peroxidation) and reduction of fall of the catalase and superoxide dismutase enzymatic activities determined in gastric mucosa [24]. Furthermore, the protective effect on mucosal cells of* Opuntia ficus-indica *cladodes and* Olea europaea *leaf extract has been already showed in* in vitro* models simulating* in vivo* condition [41].

In the present study, in subjects with GER with not severe but frequent symptoms 2 months of treatment with a medical device based on alginate/bicarbonate,* Opuntia ficus-indica* and* Olea europaea* resulted in a high symptom relief comparable with daily two months' treatment with placebo. Moreover, overall quality of life at the end of treatment is quietly different being significantly more favourable in subjects treated with the medical device. At the contrary quality of life is only lightly changed only in a reduced portion of subjects treated with placebo.

Unlike other trials published up to now on GER, antacid products PPIs and other drugs used for gastric symptoms were not admitted in this study. This was possible on the basis of the inclusion criteria designed for the recruitment of people with GER without other complications such as erosive forms.

In conclusion, our findings demonstrate that Mucosave formulation is well tolerated and highly effective in controlling symptoms associated with moderate GER. It reduces heartburn and acid regurgitation (with consequent decrease of abnormal esophageal acid exposure) by modifying the number of acid reflux episodes.

The results obtained show the protective effects of the medical device against GER and health-related quality of life. This protective action is probably related to the ability of ingredients to maintain the gastric mucosa cell membrane integrity, by their antilipid peroxidative activity, protecting against oxidative damage, and ability to strengthen the mucosal barrier, which is the first line of defense against exogenous and endogenous erosive agents.

In summary, this randomized-controlled study showed that Mucosave sachet combination reduced the scores of two questionnaires measuring symptomatology related to gastroesophageal reflux. In particular, there is evidence that the main symptoms heartburn and acid regurgitation are almost abolished and quality of life is significantly improved. To conclude, the results of this trial indicate that Mucosave sachet combination provides an effective and well-tolerated treatment for reducing the frequency and intensity of symptoms associated with gastroesophageal reflux.

## Figures and Tables

**Table 1:** Demographics and baseline characteristics of participants to the study (*N* = 118; ITT population, *N* = 126). Group 1 (*verum* 6 g/die); Group 2 (*placebo*). ITT: intention to treat.

Demographics	All the subjects	Group 1	Group 2
Age, years	49.5 ± 7.2	51.01 ± 7.6	50.05 ± 5.5
Male/female	56/62	28/31	28/31
Body mass index, kg m^2^	24.65 ± 2.9	24.37 ± 2.7	24.53 ± 3.0
Smokers	21	11	10
